# Who Is Responsible for Work–Life Balance?

**DOI:** 10.3389/fped.2015.00121

**Published:** 2016-01-19

**Authors:** Myke Drayer Federman

**Affiliations:** ^1^Pediatric Critical Care, UCLA Medical Center, Mattel Children’s Hospital, Los Angeles, CA, USA

**Keywords:** work–life balance, maternity leave, working mother, day care, gender inequality

The problem with the concept of work–life balance is that most of the onus of achieving such clarity and satisfaction is left to the employee. As a pediatric intensivist and mother of two, I am ruled by the demands and requirements imposed both by my employer and academic position and by my beautiful children. It is the culture around and perception of working mothers that needs to change – both at work and in life. This would finally allow women to find balance and promote their success in all aspects of their lives.

It seems every day there are new articles, blogs, and reports, you name it, which discuss work–life balance. Everyone has their “secrets” on how you too can achieve the perfect distribution of time, effort, and success between work and life. A quick Internet search will tell you to “Drop activities that sap your time and energy!”, “Rethink your errands!” (1), “Leave work at work!” (2), or, my favorite, “Rethink your idea of clean!” (3). Yes, a messy house is just the thing that will make me feel more balanced. Of course, there are small things each of us can do to prioritize the things that are important to us, but in order for us all, particularly working mothers, to find this elusive “balance” in our lives, it is our work environment and culture around work and life that needs to change.

There are many aspects of the general work environment in the United States that do not support the working mother. The sad state of maternity leave in this country is one of the clearest examples of how poorly we support women trying to balance work and family. The United States currently ranks 20th out of the 21 high income countries in terms of the length of protected maternity leave at only 12 weeks and, along with Oman, is one of the only two countries that does not provide paid maternity leave (4). Of course, there are exceptions in this country: Netflix recently announced it would offer unlimited paid maternity leave to its employees, but only those on its digital side, not the lower paid, more easy to replace line workers (5). Unfortunately, this minimalist approach to maternity leave and pregnancy does not stop with employee policies. One of my colleagues, a neonatologist, planned an all too brief maternity leave, but was asked 2 weeks in when she would be returning from “vacation.” Or what about the fact that our own ruling board, The American Board of Pediatrics, would not let me take the critical care board exam locally when I was 39 weeks pregnant? They insisted the pregnancy was not a disability and I would have to travel hundreds of miles away to take my exam endangering myself and my unborn daughter.

The return to work is not all that welcoming either. Exhausted, emotional and forced back to work too early, many women long to continue breastfeeding, but we find ourselves hidden in dirty bathrooms pumping in secrecy since pump rooms are not always made available for employees. Returning to work also requires finding an affordable, high quality, loving environment for the new little one who, if we were kangaroos, would not even have left the pouch yet. Finding this type of care, whether it be daycare or nanny care, is quite challenging. The cost of high quality child care is astronomical in this country and infant care is even more expensive and difficult to find. I am incredibly fortunate to have phenomenal care for my children on the campus which I work, but I could pay in-state tuition for three children here at UCLA for the same price I pay for daycare for two.

In addition to the logistical difficulty of coming back to work, most women face questions and opinions regarding their ability to commit to both work and family. In a study where fake resumes that differed only by the sex and parental status of the applicant were evaluated, women with children were seen as less competent than women without children, though men with children were seen as equally competent and more warm than men without children. These assessments led to less interest in hiring, promoting, and educating working mothers when compared to working fathers or employees without children (6). In a similar study using fake resumes in a “laboratory” setting as well as sending fake resumes to actual potential employers, mothers were rated as less competent and less committed than non-mothers, but fathers were actually seen as being more committed and were offered higher starting salaries than non-fathers (7). This aptly named phenomenon, the “Motherhood penalty” is supported by countless other studies and has been documented in many countries outside of the United States. Women face increasingly negative perceptions about their commitment and ability as they have more children, whereas men are seen in a more positive light as their family grows.

Mothers need to be supported better at work. The United States used to rank seventh in terms of the proportion of women in the workplace, but we have recently dropped to 20th, just behind Japan. The disappearance of women from the work force has the potential of reducing family standards of living and negatively affects the economy. In addition, there is mounting evidence that having a working mother has economic, educational, and social benefits for children of both sexes (8). A recent study by Kathleen McGinn from Harvard Business School showed that daughters of working mothers were more likely to be employed, had higher incomes, and were more likely to have supervisory positions than daughters of non-working mothers (9).

As a start, we need to have comprehensive policies to better support maternity leave and breastfeeding at work. Large employers should consider providing on-site daycare that is affordable and convenient. These efforts along with other initiatives to encourage work–life balance have been shown to benefit not only the employee, but the employer as well. Organizations with strong, well-established work policies demonstrate higher organizational performance, market performance, and profit-sales growth (10) and employees of such organizations have higher job satisfaction, are more likely to stay at their job and have greater pride in their organization (11).

Employer efforts would go a long way to change the perception of working mothers by recognizing the challenges of balancing work and family and having policies in place to support them. However, this likely would not be enough. Women (and men) who find themselves in leadership roles need to take advantage of their position to support other women, particularly working mothers. One study of a large law firm showed that female attorneys were more likely to be promoted and stay at the law firm if they had female partners as mentors and role models (12). Leaders should be mindful of how organizing meetings and committees may put more stress on working mothers by scheduling meetings, for example, at 7:00 a.m. or 6:00 p.m. I was once invited to join the “Women in Science” Committee in a discussion of the challenges of women in academic medicine, but the meeting was scheduled from 5:00 p.m. to 7:00 p.m. This forces working mothers to make difficult choices and to limit their involvement in committees or meetings that may be meaningful for their careers. It also forces them to make excuses as to why they cannot participate, which is often negatively perceived. As a working mother oncologist has quoted saying, “Women need to stop apologizing for wanting and needing to be with their kids in addition to fulfilling their careers and playing a role in society” (13).

## Author Contributions

MF wrote this opinion piece in its entirety.

## Conflict of Interest Statement

The author declares that the research was conducted in the absence of any commercial or financial relationships that could be construed as a potential conflict of interest.

## References

[1] Uscher J 5 Tips for Better Work-Life Balance (2011). Available from: http://www.webmd.com/women/features/balance-life?page=1

[2] Jeffries S Ten Tips for a Better Work-Life Balance (2014). Available from: http://www.theguardian.com/lifeandstyle/2014/nov/07/ten-tips-for-a-better-work-life-balance

[3] Smith J 8 Ways to Achieve Better Work-Life Balance (2013). Available from: http://www.forbes.com/sites/jacquelynsmith/2013/04/18/8-ways-to-achieve-better-work-life-balance/#2715e4857a0b1cdc41463a25

[4] Gornick J Ray R Schmitt J Parental Leave in 21 Countries: Assessing Generosity and Gender Equality. Center for Economic and Policy Research (2008).

[5] DePillis L What Netflix’s New Maternity Leave Policy Doesn’t Tell Us About Inequality (2015). Available from: https://www.washingtonpost.com/news/wonk/wp/2015/09/01/netflixs-much-criticized-maternity-leave-policy-is-actually-pretty-rare/

[6] Cuddy A Fiske S When professionals become mothers, warmth doesn’t cut the ice. J Soc Issues (2004) 60(4):701–18. 10.1111/j.0022-4537.2004.00381.x

[7] Correll S Bernard S Paik I Getting a job: is there a motherhood penalty? Am J Sociol (2007) 112:1297–338. 10.1086/511799

[8] Miller CC Mounting Evidence of Advantages for Children of Working Mothers. New York Times, May 15 (2015).

[9] Nobel C Kids Benefit from Having a Working Mom (2015). Available from: http://hbswk.hbs.edu/item/kids-benefit-from-having-a-working-mom

[10] Perry-Smith J Blum T Work-family human resource bundles and perceived organizational performance. Acad Manag J (2000) 43(6):1107–17. 10.2307/1556339

[11] Kenexa Research Institute. Work Trends Survey (2007). Available from: www.hr.com

[12] McGinn KL Milkman KL Looking up and looking out: career mobility effects of demographic similarity among professionals. Org Sci (2013) 24(4):1041–90. 10.1287/orsc.1120.0778

[13] Belkin L Should Women Be Doctors? New York Times, June 13 (2011).

